# Application of pulsed electric field technology to skin engineering

**DOI:** 10.3389/fbioe.2024.1386725

**Published:** 2024-04-16

**Authors:** C. Berry-Kilgour, L. Wise, J. King, I. Oey

**Affiliations:** ^1^ Department of Pharmacology and Toxicology, School of Biomedical Sciences, University of Otago, Dunedin, New Zealand; ^2^ Department of Food Sciences, University of Otago, Dunedin, New Zealand; ^3^ Riddet Institute, Palmerston North, New Zealand

**Keywords:** pulsed electric field (PEF), tissue engineering, skin cell culture, biomaterial preparation, skin substitutes

## Abstract

Tissue engineering encompasses a range of techniques that direct the growth of cells into a living tissue construct for regenerative medicine applications, disease models, drug discovery, and safety testing. These techniques have been implemented to alleviate the clinical burdens of impaired healing of skin, bone, and other tissues. Construct development requires the integration of tissue-specific cells and/or an extracellular matrix-mimicking biomaterial for structural support. Production of such constructs is generally expensive and environmentally costly, thus eco-sustainable approaches should be explored. Pulsed electric field (PEF) technology is a nonthermal physical processing method commonly used in food production and biomedical applications. In this review, the key principles of PEF and the application of PEF technology for skin engineering will be discussed, with an emphasis on how PEF can be applied to skin cells to modify their behaviour, and to biomaterials to assist in their isolation or sterilisation, or to modify their physical properties. The findings indicate that the success of PEF in tissue engineering will be reliant on systematic evaluation of key parameters, such as electric field strength, and their impact on different skin cell and biomaterial types. Linking tangible input parameters to biological responses critical to healing will assist with the development of PEF as a sustainable tool for skin repair and other tissue engineering applications.

## 1 Introduction

Injuries, diseases, or infections can lead to tissue damage, which undermines the integrity and function of the affected tissue. The body possesses innate mechanisms for repair to maintain tissue homeostasis, but substantial damage can delay or impair this restoration process (Demidova-Rice et al., 2012a; Berry-Kilgour et al., 2021). When skin tissue is damaged, the occurrence of pathological healing manifesting in chronic non-healing wounds or abnormal scarring has significant implications for patient outcomes (Kapp and Santamaria, 2017; Nussbaum et al., 2018). Following cutaneous wounding, a coordinated effort involving cells, growth factors, cytokines, and extracellular matrix (ECM) contributes to a phasic healing process characterised by homeostasis, inflammation, re-epithelialisation, and remodelling (Berry-Kilgour et al., 2021). In instances of skin loss with large surface area, restoration of function is slow and relies on secondary-intention healing, leaving the patient at risk for infection, prolonged hospital stays, and reduced quality of life (Sussman and Bates-Jensen, 2007; Chetter et al., 2019).

Skin substitutes and functional living grafts have the potential to replace traditional grafting approaches for burns or traumatic wounds and may even advance to the development of more complex, full-thickness constructs for deep chronic wounds. Achieving this goal relies upon the application of tissue engineering techniques. Tissue engineering is a multifaceted field which relies on two key factors: the successful proliferation and differentiation of the desired cell type/s, and the careful selection of an ECM-mimicking biomaterial to provide structural support for the developing construct (Sharma et al., 2019). The resulting tissue must possess characteristics mirroring those of the target tissue, including the correct anatomical features, vascularisation, porosity, elasticity, stiffness, and functionality (Sharma et al., 2019; Lanza et al., 2020).

To make engineered tissue constructs relevant to the target tissue, the correct cell types must be selected, and these cells must be directed to proliferate, migrate, and differentiate according to the desired phenotype (Sharma et al., 2019). In skin engineering, there has been a heavy focus on the epidermis - the outer keratinocyte-based layer, and the lower dermis, which predominately contains ECM-secreting fibroblasts (Dearman et al., 2021). Often neglected is the hypodermis, the third and innermost layer, containing adipose and immune cells, but recently its importance in skin homeostasis has been acknowledged and tri-layer skin constructs are becoming more popular (Workman et al., 2023). Generating the correct tissue type typically involves the delivery of exogenous growth factors and signalling molecules to the cultured cells, which can be challenging when applied to tissue constructs due to reduced stability and high production cost (Sharma et al., 2019).

Isolation and preparation of the chosen biomaterials must consider the biocompatibility of the end product. The biomaterials must be sterile and capable of interacting with the cellular environment to enhance development, healing, or function of a tissue (Bianchera et al., 2020). In the development of skin substitutes, selection of these biomaterials should consider the multi-layered structure of the skin and there should be an understanding of how structural requirements may change across the dermis, epidermis, and hypodermis (Abdo et al., 2020). The environmental impact of biomaterial processing also needs to be considered. While biopolymers and natural polymers present environmental advantages over synthetic polymers (Gowthaman et al., 2021), they still come with their limitations. For example, extraction of collagen and cellulose fibres typically involves high quantities of acid and alkaline solutions for hydrolysis (Radotić and Mićić, 2016; Matinong et al., 2022). In turn, production of scaffolding from these materials and their subsequent testing requires substantial financial investment (Hollister, 2009; Kim et al., 2019). As the current methods for tissue engineering are both environmentally and financially costly, it is critical that economical solutions are identified.

Pulsed electric field (PEF) processing is an environmentally sustainable method involving application of an electrical field to material (Oey et al., 2022). This technique has been widely used in medical and non-medical applications, such as food production, to modify the microstructure and functionality of liquid, semi-solid and solid biological materials (Arshad et al., 2020), including 3D tissues such as meat (Alahakoon et al., 2017; Karki et al., 2023), fruit and vegetables (Alpos et al., 2022; Leong et al., 2022). Treatment of liquids has focused largely on microbial inactivation (Sharma et al., 2014), but more recently has gained interest in enhancing the extraction of constituents, such as oil, from plants for biomedical development (Zbinden et al., 2013; Pataro et al., 2017; Ranjha et al., 2021), and colour and flavour compounds from grapes for wine production (Puértolas et al., 2010; Arcena et al., 2021). Pulse electric fields have also become increasingly utilised in the biomedical space, with established roles in the non-thermal ablation of cancerous tissue, in electrochemotherapy by facilitating the transport of drugs across cell membranes and transdermal barriers, and in gene therapy and DNA vaccination through gene electrotransfer to cells or tissues, including cutaneous wounds (Prausnitz and Langer, 2008; Thomson et al., 2011; Yarmush et al., 2014; Gibot and Rols, 2016). Further the application of PEF to the skin is supported by its transepidermal potential which forms during development, as this generates endogenous electric fields upon injury which direct the migration of numerous skin cells to facilitate wound closure (Reid and Zhao, 2013; Abe and Nishizawa, 2021). This has led to exogenous electrical fields being increasingly applied to promote healing of skin wounds. These intriguing uses of PEF raise questions as to how PEF is best utilised in the field of skin engineering, through application to skin cells or their scaffolds, and how the effects of PEF can be controlled for predictable and tailorable biological outcomes. Therefore, this review will introduce the key principles of PEF technology and discuss how it has and could be applied in the context of skin engineering.

## 2 Current approaches to skin engineering

### 2.1 Skin grafting

The classical approach for treating skin loss due to disease or injury is autologous grafting (Table 1), where skin is removed from the patient at an alternative site to assist with closure of the primary wound (Herskovitz et al., 2016). Grafts can be classified as full-thickness or split-thickness, where full-thickness includes the entirety of the epidermis and dermis, and split-thickness include epidermis and only part of the dermis (Herskovitz et al., 2016). The clinical gold standard remains an autologous, split-thickness skin graft. Despite their popularity, skin grafts can be problematic because they involve production of another wound at a secondary site, placing further healing burden on the patient (Dearman et al., 2021). Skin grafts come with additional risks, including rejection, infection, and seroma (Kohlhauser et al., 2021). The donor site is particularly problematic, with moderate to high reported incidences of pain, hypertrophic scarring incidence up to 28%, infection, and reduced quality of life (Asuku et al., 2021).

**TABLE 1:** Current approaches to skin substitution and their limitations.

Therapeutic approach		Example products	Limitations	References
Skin Grafts	Autologous or allogenic	Full-thickness or split thickness	Requires secondary wound site	Herskovitz et al. (2016), Asuku et al. (2021), Dearman et al. (2021), Kohlhauser et al. (2021), Schlottmann et al. (2021)
			Prone to infection, inconsistent healing, scarring	
			Immune rejection	
Acellular skin substitutes	Decellularised human matrices	AlloPatch	Do not function as complete dermal replacements–still requiring skin grafting	Brigido (2006), Mofid and Singh (2009), Wainwright and Bury (2011)
		Alloderm	Narrow patient window	
		GraftJacket	High cost	
			Inconsistent decellularisation efficacy Inconsistent recellularisation capacity Storage needs	
	Decellularised animal matrices	Matristem UBM and derivatives (porcine urinary bladder)	Ethical and accessibility concerns	Brown-Etris et al. (2019), Amin et al. (2022), Hill et al. (2022), Smith et al. (2022)
		Architect collagen matrix	High cost	
		Endoform (ovine stomach) PriMatrix (foetal bovine dermis)	Inconsistent decellularisation efficacy	
		Oasis (porcine small intestinal mucosa)	Storage needs	
		Kerecis Omega3 (fish skin)		
	Reconstituted animal matrices	Bio-ConneKt (collagen)	Narrow patient window	Agosti and Chandler (2015), Sharma et al. (2023)
		Integra (collagen/GAG)	Immunogenicity/hypersensitivity	
		Helicoll (collagen)	High cost	
		Excellagen gel (collagen)	Storage needs	
	Decellularised human placental membranes	Allowrap	Adverse reactions	Protzman et al. (2023)
		Neox	Inconsistent decellularisation efficacy	
		Biovance		
		AmnioExcel		
	Engineered matrices	Hyalomatrix (Hyaluronic acid)	Cannot be used in third-degree burns	Motolese et al. (2013), Agosti and Chandler (2015), Sharma et al. (2023)
		Restrata (polyglactin)	Adverse reactions (inflammation, rejection)	
		Integra Bilayer (collagen/GAG)		
		Integra Regeneration (collagen/GAG)		
		Aquacel (CMC)		
		NovoSorb BTM (polyurethane)		
Cellular skin substitutes	Human placental allografts	Affinity	Variability associated with donors	Nejad et al. (2021), Protzman et al. (2023)
		FloGraft	Complex manufacturing conditions	
		Grafix	High cost	
			Complex storage conditions	
			Immune rejection	
			Do not always act as total skin replacements	
	Engineered cell-laden matrices (synthetic, animal, human)	Dermagraft (polyglactin + fibroblasts)	High cost	Still et al. (2003), Zaulyanov and Kirsner (2007), Hart et al. (2012), Sharma et al. (2023)
		Orcel (collagen + fibroblasts and keratinocytes)	Complex storage conditions	
		SkinTE (patient skin)	Immune rejection	
		Theraskin (human skin allograft)	Do not always act as total skin replacements	
		Apligraf (collagen matrix + fibroblasts)		

In cases where sufficient tissue cannot be provided by the patient, allogenic grafts, where the graft is harvested from an alternative donor, can be used (Table 1) (Schlottmann et al., 2021). Allogenic grafts come with a greater risk of immunogenicity and subsequent graft rejection due to their foreign nature (Schlottmann et al., 2021). The healing outcomes following skin grafts are not always consistent, particularly following allogenic grafting (Schlottmann et al., 2021).

### 2.2 Acellular skin substitutes

In the absence of autologous or allogenic skin grafting, skin substitutes can be used (Table 1) (Halim et al., 2010). These do not necessarily have to be human-derived but must be biocompatible and capable of supporting the wound as it heals. Using biomaterials as a base to construct porous or fibrous scaffolds is one strategy, producing 3-dimensional (3D) skin substitutes which provide structural support to the wound (Sheikholeslam et al., 2018). Biomaterials can be isolated from natural and synthetic sources (Lee et al., 2018). Biomaterial-based scaffolds can be produced by a number of techniques including self-assembly (Webber and Pashuck, 2021), electrospinning (Rahmati et al., 2021), 3D printing (Richards et al., 2013), and decellularisation (Neishabouri et al., 2022). Porosity of the scaffold is critical, because this can alter cell interactions and change the capacity for the scaffold to transfer nutrients and oxygen (Loh and Choong, 2013). The ECM that surrounds and supports skin cells *in vivo* is a complex arrangement of collagen, elastin, and glycosaminoglycans (GAGs), and each of these play a role in maintaining skin homeostasis and structural support, as well as contributing to the healing process following skin injury (Rousselle et al., 2019). Therefore, constructing skin substitutes from human ECM components, e.g., collagen, is a logical approach. Collagen molecules are triple helices that assemble into fibrils, a few hundred nanometres in diameter, which form basket weave structures within native skin (Fratzl, 2003). Mammalian collagen (e.g., from bovine, porcine, ovine sources) can be formulated into porous scaffolds by 3D-printing, and fibrous scaffolds by self-assembly or electrospinning (Sheikholeslam et al., 2018). As a biomaterial, collagen can interact with cells and the ECM of the recipient to promote cellular proliferation (Parenteau-Bareil et al., 2010). Mammalian collagen scaffolds include Bio-ConneKt and HeliColl, among others (Table 1) (Sharma et al., 2023). Unfortunately, mammalian collagen has been associated with immunogenicity following implantation, with ethical controversy complicating its use (Parenteau-Bareil et al., 2010).

Decellularisation allows for preservation of the ECM structure as a whole, isolating all of its components (collagen, elastin, GAGs) while removing potentially immunogenic cellular components (Crapo et al., 2011). Human and animal-derived decellularised matrices are commercially available for wound healing indications (Table 1), including Alloderm^®^ and GraftJacket™ from donor cadaver skin (Dussoyer et al., 2020) and Matristem urinary bladder matrix (UBM)™ from porcine bladder tissue (Kimmel et al., 2010). Placement of these structures on the wound allow for migrating cells from the neighbouring tissue to populate the scaffold as healing occurs (Wei et al., 2002). However, these structures are associated with complications. In addition to continued ethical criticism, mammalian acellular matrices can be inconsistent in structure and recellularisation capacity, based on the features of the donor (Gilpin et al., 2014; Johnson et al., 2016). Additionally, mammalian ECM components or ECM fragments generated during the decellularisation process are capable of inducing immune reactions within the recipient (Allaire et al., 1997; McQuitty et al., 2020). Production of these fragments can be linked to the chemical-heavy approaches currently used for decellularisation (Crapo et al., 2011).

Biologically inert materials are potentially advantageous over biologically active materials because they reduce the opportunity for immunogenicity while still providing the structural support required for healing (Hickey and Pelling, 2019). For example, cellulose is an inert biomaterial derived from bacterial and plant sources and is associated with good biocompatibility with wound healing cells (Modulevsky et al., 2014; Modulevsky et al., 2016; Koivuniemi et al., 2020). While cellulose molecules differ from those of collagen, in that they consist of chains with repeats of two sugar rings, they also naturally exist as thin, spiraling microfibrils, but contain nano-to micro-metre crystals depending on their source (Fratzl, 2003). Hydroxyl groups present on cellulose chains allow for cell adhesion and cellulose has high stability and tensile strength (Naomi et al., 2020). Cellulose can be extracted as crystals, fibres, or decellularised scaffolds, formulated as hydrogels, or used as bioink for 3D-printing (Sharip and Ariffin, 2019). Carboxymethylcellulose, a form of modified cellulose, is a well-characterised biomaterial in the wound space and has been formulated into products such as the AquaCel^®^ Hydrofiber dressing (Table 1) (Williams, 1999; Guest and Ruiz, 2005). Other biologically inert products for wound repair include the synthetic polyurethane scaffold Novosorb^®^ biodegradable temporising matrix (BTM), which has been highly successful in the treatment of complex wounds (Table 1) (Li et al., 2021). The production of these materials generally requires chemically-intensive processes, which can reduce their environmental sustainability (Radotić and Mićić, 2016; Gowthaman et al., 2021; Khan et al., 2022).

### 2.3 Cellularised skin substitutes

Incorporating cells into skin substitutes allows for synthesis of living grafts, which not only provide structural support to wounds, but contain functional skin cells that can contribute to the healing process (Table 1). Such cells include keratinocytes, fibroblasts, and endothelial cells. Keratinocytes are found throughout the epidermal layer of the skin where they create four to five differentiated layers from the deepest *stratum basale* to the outer *stratum corneum* (Montagna, 2012)*.* The highly replicative keratinocytes within the *stratum basale* are attached to a collagen-based basement membrane below which lies the dermis. As keratinocytes transition through the epidermal layers, they increase in keratin and lipid production, then flatten and die to create the dry, protective barrier of the *stratum corneum*. Also found within the epidermis are melanocytes, Merkel cells and Langerhans cells, which respond to ultraviolet radiation, physical and microbial cues, respectively. Below, the dermis is heavily populated with fibroblasts, responsible for secreting collagen, elastin and GAGs which form the surrounding ECM and provide structure, tensile strength and elasticity, and endothelial cells, which comprise the vasculature and control oxygen and nutrient supply (Montagna, 2012). The dermal layer also contains phagocytes and lymphatic vessels critical for the response to microbial breaches of the skin. The innermost hypodermis, or subcutaneous layer, comprised of well vascularised, adipose tissue within loose ECM, which provides insulation and cushioning. The skin also comprises a number of accessory organs, including sebaceous glands, hair follicles, and nervous innervation (Montagna, 2012; Abdo et al., 2020). The maintenance and regeneration of cells and organs within the skin is directed by growth factors and signalling molecules, which must be supplied exogenously when constructing skin substitutes (Sharma et al., 2019).

The most simplified version of living grafts are cell sheets that secrete their own ECM. These cells are generally isolated from skin biopsies, from the patient or healthy donors. The isolated cells are expanded *in vitro* through the addition of growth supplements*,* then applied as thin films of confluent cell layers (Chaudhari et al., 2016). Further, autologous self-assembled skin substitutes (SASS) have been created that allow for replacement of both the dermis and epidermis in a single procedure. An acellular dermis construct is prepared through self-synthesis of a collagen-rich ECM by the patient’s fibroblasts. The patient’s keratinocytes are then cultured on the dermal construct to form a stratified epidermis, which is transplanted onto full-thickness burns or wounds (Athanasiou et al., 2013; Dagher et al., 2023). In addition, spray suspensions of keratinocytes and fibroblasts have been used successfully to apply cells in a thin layer across the surface of the wound, assisting with re-epithelialisation (Motamedi et al., 2021). Cell sprays have also been used in tandem with other skin substitutes, like skin grafts and biomaterial scaffolding (Motamedi et al., 2021).

Porous scaffolds seeded with cells provide structurally appropriate materials for more substantial wounds with deeper skin loss. For example, Dermagraft^®^, a polyglactin mesh seeded with fibroblasts, which is approved for use in diabetic foot ulcers (Table 1) (Hart et al., 2012). Fibroblasts populating the matrix secrete their own ECM and incorporate with the wound below to increase the rate of healing (Hart et al., 2012). By producing multi-dimensional scaffolds, it is also possible to seed multiple cell types within layers to replicate the multi-layered structure of native skin. For example, Orcel^®^ is a collagen-based sponge seeded with keratinocytes in an upper gel layer and fibroblasts in a lower porous layer to replicate the epidermis and lower dermis (Table 1) (Still et al., 2003). This product is U.S. Food and Drug Administration-approved for the treatment of burn autograft sites and acute surgical wounds. Unfortunately, many of these engineered skin substitutes, both acellular and cellular, are not yet functioning as total skin replacements. In many cases, they still need to be applied in combination with a split thickness skin graft or acellular fillers (Halim et al., 2010; Wainwright and Bury, 2011). They also have a limited patient window, often only appropriate for wounds free of infection, with good vascularisation (Wainwright and Bury, 2011).

### 2.4 Challenges with current skin engineering approaches

Beyond the biological level, there are other spaces for improvement, as the techniques used to produce engineered skin substitutes need to be scalable to the industry level (Dearman et al., 2021). Additionally, these products need to be financially accessible to patients. Many skin substitutes are associated with high cost (Table 1): OrCel^®^, a bilayer dermal substitute, costs an estimated $27.78USD per cm^2^, and Alloderm^®^ a decellularised human dermal matrix, costs an estimated $29.68USD per cm^2^ (Mofid and Singh, 2009; Pourmoussa et al., 2016). High cost of skin substitutes remains a limitation not only to patient accessibility, but to scalability and manufacturing (Halim et al., 2010; Pangarkar et al., 2010). It is also becoming more important that tissue constructs are prepared using environmentally friendly and sustainable methods (Mahmud et al., 2023; Benko and Webster, 2023; D’Elía et al., 2023). The use of naturally-derived biomaterials with sustainable sources such as marine collagen and cellulose are compelling, but the extraction and production of these biomaterials remains a chemically-intensive process (Samavedi et al., 2014; El Knidri et al., 2018; Khan et al., 2022; Saji et al., 2022). The use of growth factors and signalling molecules to support cellular proliferation and differentiation within engineered constructs remains a resource-intensive and costly addition and alternative approaches to support cellular function are needed (Ren et al., 2020).

## 3 An overview of the principle of PEF

**FIGURE 1:**
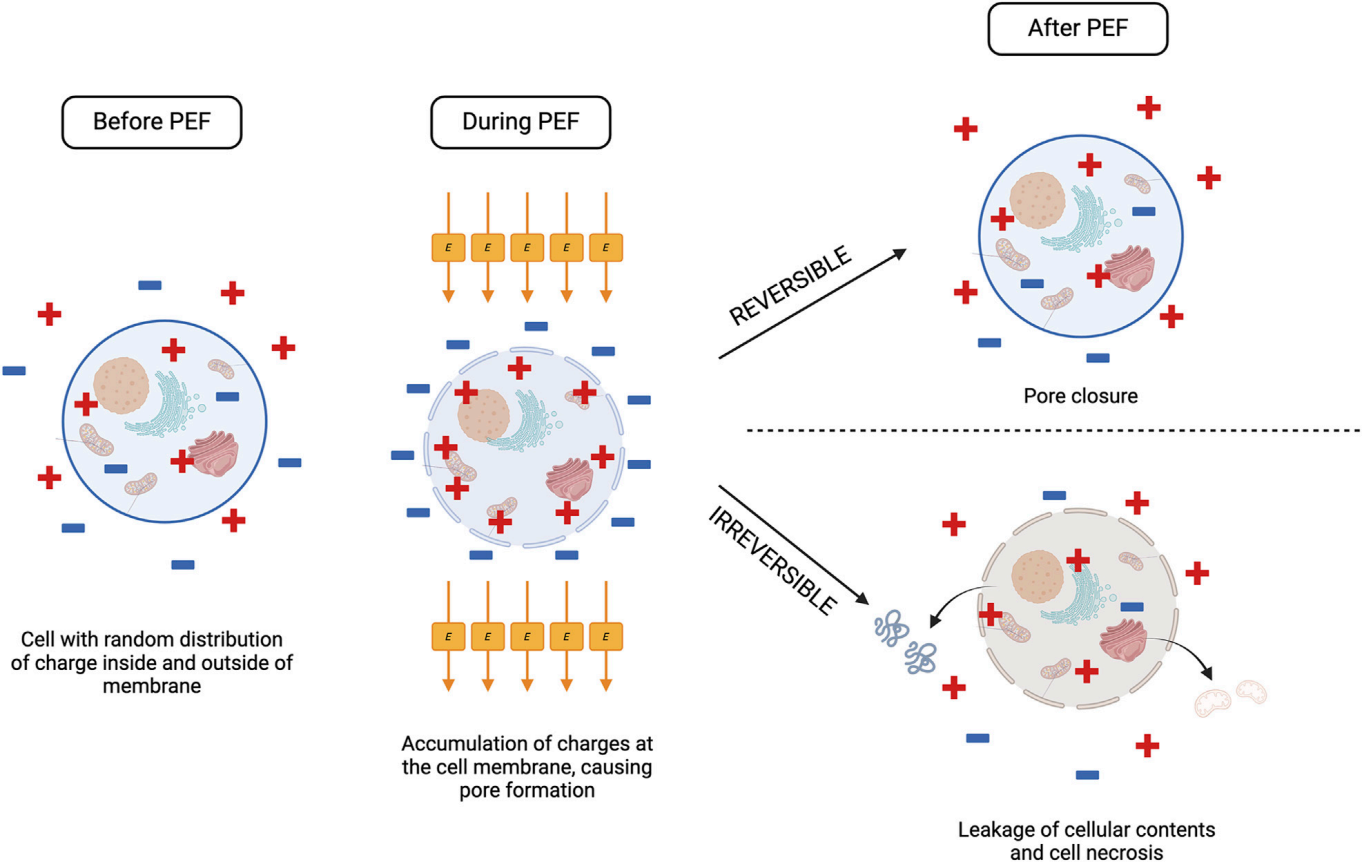
The effect of PEF processing on a cell. When a cell is exposed to PEF, the electric field promotes accumulation of positive and negative charges at the cell membrane, disrupting the transmembrane potential and causing pore formation. Depending on the electric field strength (*E*) reached, the cell can respond one of two ways following PEF treatment. If *E* does not exceed the critical threshold for the cell, pores will close, and the cell returns to normal function (reversible electroporation). If *E* exceeds the critical threshold, electroporation becomes irreversible, and there is leakage of the cell contents from the permanently formed pores as the cell undergoes necrosis. Created with BioRender.com.

Pulse electric field is a process by which short, repetitive pulses are transferred to a target tissue via electrodes at a predefined voltage, frequency, pulse duration, and exposure time (Figure 1) (Oey et al., 2022). Typically, the sample is placed in a chamber with two opposing metal electrode plates on either side, which direct the electrical field through the tissue (Töpfl, 2006). The formation of an electric field (*E*) around the target tissue leads to the formation of pores in the cellular membrane due to alteration of the transmembrane potential. This pore formation, known as electroporation, can be reversible, where the pores close following neutralisation of the electric field, or irreversible, whereby permanent pore formation causes leakage of the cellular contents and necrosis of the cell (Edd et al., 2006; Lee et al., 2010).

A reversible or irreversible outcome is dependent on the electric field intensity applied to the target tissue, which above a certain transmembrane potential threshold will result in irreversible pore formation (Rols and Teissié, 1990). This critical electric field strength will differ based on the target tissue and is heavily dependent on conductivity and cell size. As plant and animal cells are larger they require a lower critical electric field strength compared to smaller microbial cells (Bhat et al., 2019).

## 4 Using PEF technology to direct cell fate

**FIGURE 2:**
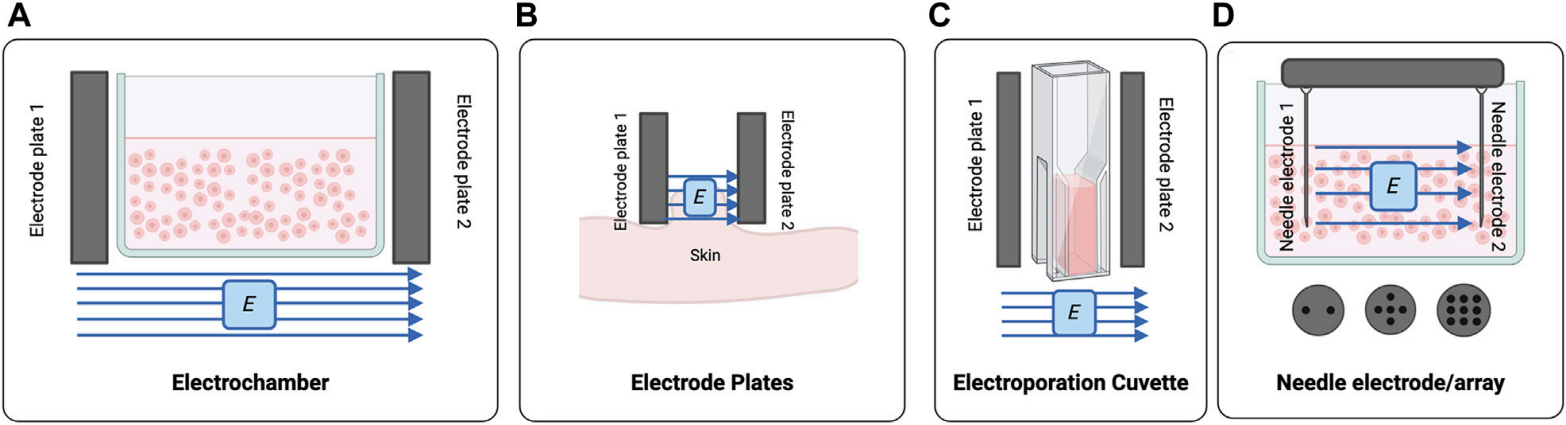
The PEF systems used for processing of cells and tissues **(A)** Classical electrochambers can be modified to house cell suspensions which can be treated using opposing electrode plates. **(B)** Electrode plates are positioned perpendicular to the target tissue. **(C)** Electroporation cuvettes are a derivation of the electrochamber, generally with smaller volumes. **(D)** Needle electrodes and/or electrode arrays can be used to treat multi-well plates or incorporate more than two electrodes. Created with BioRender.com.

Pulse electric field systems for cell applications have been developed for *in vitro* and *in vivo* applications (Figure 2). Control of cell fate is a critical part of producing successful tissue constructs (Demidova-Rice et al., 2012b; Lanza et al., 2020; Battafarano et al., 2021) and application of an exogenous electric field to cells using PEF has the capacity to influence their migration, proliferation, and functionality (Ren et al., 2019; Gouarderes et al., 2020; Gouarderes et al., 2022). PEF treatment of skin cells has focused largely on epidermal and dermal cell types including fibroblasts (Liu et al., 2018; Nguyen et al., 2018; Das et al., 2020; Gouarderes et al., 2020) and keratinocytes (Ren et al., 2019; Das et al., 2020) (Table 2).

**TABLE 2:** Application of PEF to control cellular healing responses.

Tissue	Cell type(s)	Model	Equipment	Electric field strength (V/cm)	Pulse shape	Pulse duration	Pulse frequency (Hz)	Pulse exposure/number	Outcome	References
Skin cells (*in vitro*)	Human dermal fibroblast, HaCaT keratinocyte	N/A	Needle electrodes	100–600	Not stated	70 µs	1	90 pulses	Enhanced closure rate for fibroblasts and keratinocytes in a scratch wound assay at 500 V. Conditioned media enhanced closure of untreated cells in a wound scratch assay	Das et al. (2020)
	Human dermal fibroblast	N/A	Electrode plates in Petri dish or 96-well plate	200–800 (A)	Square	100 µs (A)	1	8 pulses (A) 10 pulses (B)	No significant effect on fibroblast migration. Cytotoxicity at high *E* (800V/cm or 300V/cm). Increased CTGF, VEGF-A, PDGF-A, TGF-α, and reduced TGF-β expression	Gouarderes et al. (2020)
				50–300 (B)		5 ms (B)				
	Primary mouse keratinocyte	N/A	Electrochamber with two parallel electrode plates	0.05–0.25	Square	600 µs– 600 m	0.1–1,000	3 h	Migration and speed of keratinocytes increased relative to *E* but not pulse frequency	Ren et al. (2019)
	Primary mouse fibroblast	N/A	Electrochamber with two parallel electrode plates	0, 0.1, 0.2, 0.3	Square	0.01, 1, 100, 5000 s	100, 1, 0.01, 0.0002	4 h	Fibroblast alignment and contracture enhanced in a frequency-dependent manner	Liu et al. (2018)
	Human umbilical vein endothelial cell	N/A	Two titanium electrode plates	0.081, 0.162	Not stated	2 m	0.6, 1.2	48 h	Proliferation increased with *E*, but diminished after 48 h. Voltage had no effect	Abasi et al. (2023)
Skin constructs (*in vitro*)	Human dermal fibroblast	Self-assembly, anchoring paper	Two stainless steel electrodes, 0.8 cm gap	600 (LP)	Square	5 m (LP)	1	3 pulses (LP)	Transient decrease in TGF-β1 and collagen production and increase in MMP activity with LP.	Gouarderes et al. (2022)
				1,000 (SP)		100 µs(SP)		8 pulses (SP)		
	Human dermal fibroblast, HaCaT keratinocyte	Derm-ACELL^®^ grafts	Electroporation cuvette, 0.2 cm gap	300	Not stated	150 ms	Not stated	8 pulses	Luciferase expression persisted for 1 week after gene electrotransfer of recellularised skin constructs	Bulysheva et al. (2016)
Skin (*in vivo*)	N/A	Human diabetic ischaemic ulcer	NewHealth 9,000 apparatus, mat on which patient can sit or lie	2000–9,000	Not stated	Not stated	50	40 min, 13 sessions, thrice weekly, alternate days	Reduced ulcer area and pain scores compared to untreated controls. Improvements in % SpO_2_, diastolic blood pressure and heart rate	Liani et al. (2014)
	N/A	Human pressure ulcer	Foil electrode over ulcer area	100–175	Not stated	Not stated	120	45, 60, 120 min, daily, for up to 5 weeks	Wound surface area at 3 and 5 weeks increased in 60- and 120-min treatment groups compared to 45-min and control groups	Ahmad (2008)
	N/A	Human graft post- burn	Neurodyn High Volt^®^	>100	Not stated	15 µs	100	50 min	Reduced pain, improved re-epithelialisation, dressing detachment, and scar quality scores with treatment compared to untreated controls	Gomes et al. (2018)
	N/A	Human skin (healthy)	Fenzian System	20–80	Not stated	1.6 m	60	30 min on days 7, 10, 12, 14 or 14, 17, 21, 24	Reduced wound volume, surface area and diameter all reduced on day 10. Increased blood flow, PlGF and VEGF-A expression	Ud-Din et al. (2015)
	N/A	Rabbit skin flap and ischaemic hind limb	Electroporation cuvette, 0.4 cm gap	30,000	Not stated	300 ns	Not stated	5 pulses	Reperfusion of skin flap wounds enhanced compared to saline on days 3 and 21. No effect on reperfusion of hind-limbs, but increased endothelial cell, collagen, and VEGF staining	Hargrave and Li (2015)

PEF treatment of cell suspensions or monolayers has been successfully executed using modified electrochambers (Hess et al., 2012; Liu et al., 2018; Ren et al., 2019) or electroporation cuvettes comprised of two opposing electrode plates on a cuvette (Hargrave and Li, 2012; Hargrave and Li, 2015) (Figure 2). Needle electrodes composed of two opposing needle electrodes or multi-needle arrays have also been arranged to deliver electric fields to multi-well plates (Figure 2) (Basu et al., 2014; Chang et al., 2017; Das et al., 2020).

Enhanced proliferative and migratory responses of fibroblasts in 2D culture following PEF treatment was observed in several studies (Table 2) (Das et al., 2020; Gouarderes et al., 2020). When embedded in 3D collagen lattices, PEF-treated fibroblasts had enhanced alignment and contractility (Liu et al., 2018; Gouarderes et al., 2022). Studies investigating the effect of PEF on keratinocytes have been limited to 2D culture and showed variable effects on their migration (Ren et al., 2019; Das et al., 2020).

Vascularisation of 3D tissue constructs is an ongoing limitation to skin engineering, where implementing a functional vasculature with transfer of oxygen and nutrients becomes increasingly difficult with increasing construct size and thickness. The effect of PEF on vascular cells is limited (Table 2), but one study showed that PEF treatment enhanced the proliferation of human umbilical vein endothelial cells (HUVECs) in 2D culture, and that this effect persisted for 48 h post-treatment (Abasi et al., 2023).

The effect of PEF on engineered skin constructs has been investigated in limited studies. When applied to a dermal substitute *in vitro,* PEF was able to modulate the surrounding ECM to promote an anti-fibrotic phenotype with reduced collagen and increased matrix metalloproteinase (MMP) production (Table 2) (Gouarderes et al., 2022). Further, electrotransfer and expression of a luciferase gene was achieved in a DermACELL^®^ grafts recellularised with *in vitro* (Bulysheva et al., 2016).

*In vivo,* the role of PEF in improving healing outcomes has been explored to a greater extent (Gibot and Golberg, 2016). PEF has successfully been applied *in vivo* to wounded skin in clinical patients with favourable outcomes on wound repair (Table 2) (Ahmad, 2008; Liani et al., 2014; Ud-Din et al., 2015; Gomes et al., 2018). The PEF systems used in these clinical cases include electrode clamps (Mir et al., 1991; Steinstraesser et al., 2014; Golberg et al., 2018; Song et al., 2021), foil electrodes overlaying the wound surface (Ahmad, 2008), electrode plates bandaged to the wound periphery as in the Neurodyn High Volt^®^ system (Gomes et al., 2018) or electrode mats such as the NewHealth 9,000 (Liani et al., 2014). These systems could certainly be utilised in tissue engineering applications, where skin tissue constructs are used as engineered grafts. Across these studies, investigators reported faster re-epithelialisation and wound closure of both acute and chronic wound types (Ahmad, 2008; Liani et al., 2014; Ud-Din et al., 2015; Gomes et al., 2018). In a study of acute wounds, Ud-Din et al reported enhanced expression of the pro-healing growth factors vascular endothelial growth factor (VEGF)-A and placental growth factor (PLGF) (Ud-Din et al., 2015). It should be noted that these pro-healing effects are most likely related to electrotaxis, a phenomenon where application of electric fields can stimulate migration and proliferation of cells, as well as enhance expression of signalling molecules (Cortese et al., 2014). Thus, the electric fields applied to these wounds may not exceed the critical threshold for electroporation, and it is yet to be elucidated whether electroporation, rather than simply application of an electric field, has any effect on healing outcomes *in vivo*. Distinguishing between electrotactic and electroporation effects will be important in understanding how PEF might be applied to different stages of skin engineering.

## 5 Using PEF technology for biomaterial preparation

Biomaterials remain a critical feature of any tissue construct, and these materials must be sterile, biocompatible, and mechanically suitable to the desired tissue (Lee et al., 2018; Bianchera et al., 2020). The use of PEF has been tested in various biomaterial fabrication processes (Table 3).

**TABLE 3:** Application of PEF for biomaterial preparation.

Purpose	Material	Equipment used	Electric field strength (kV/cm)	Pulse shape	Pulse duration	Pulse frequency (Hz)	Pulse number	Outcome	References
Sterilisation	Collagen gels	Electrochamber with two opposing plate electrodes	12–45	Square	1 µs	0.2 or 1	10, 20, 50, 100	*E. coli* inactivated in gels at low densities (<10^3^ CFU/mL), with greatest inactivation at highest *E* (45 kV/cm)	Griffiths et al. (2008)
	Collagen gels	Electrochamber	30–60	Square	1 µs	1	100	Sterilisation did not affect growth of osteoblasts on gels compared to non-treated controls	Smith et al. (2009)
	Osteoblasts								
Modification of structure	Sodium alginate bioink	PEF-assisted printing	2.5 or 3.0	Not stated	Not stated	150, 160, 170	Not stated	Frequency reduced droplet size and distance, but reduced consistency. Voltage increased droplet diameter and distance, and increased consistency. Pulse width had no significant effect	Rahman et al. (2021)
	Zein, chitosan, and poly (vinyl alcohol) (ZCP) film	Electrochamber with two opposing plate electrodes	0.9, 1.6, 2.4, 3.4	Square	32 µs	10, 50, 100, 220	Varied	At *E* > 2.4 kV/cm, particle size increased, but viscosity and loss modulus were reduced. Optimised tensile strength was achieved at 3.4 kV/cm, 50 Hz and 100 kJ/kg	Giteru et al. (2020)
Decellularisation	Rat carotid artery *in vivo*	Two electrode plates clamped either side of the vessel	1.75	Not stated	100 µs	1 or 4	90	Artery showed cell and nuclear loss in histological sections after 5 days. Effect sustained to 7 days with the 1 Hz-treated vessels, but cell repopulation was observed in 4 Hz-treated vessels	Phillips et al. (2010)
	Porcine liver *ex vivo*	Two electrode plates clamped either side of the liver	≤1	Square	100 µs	0.25, 0.5, 1.0 or 4.0 Hz	99	Visible lesions induced, with cell loss in histological sections. Largest lesion formed at 0.95 kV/cm and 1.0 Hz	Sano et al. (2010)

One such application of PEF, is in the production of decellularised biomaterials. This has been explored by a few studies as an alternative to classical chemical-based decellularisation methods (Table 3) (Phillips et al., 2010; Sano et al., 2010). Pulsed electric field processing allowed for non-chemical decellularisation of living tissues by targeting specific tissue regions using clamps or electrode plates and leaving surrounding tissue unharmed. Targeted application of an electric field to mammalian tissue was utilised both *in vivo* (rat carotid artery) (Phillips et al., 2010) and *ex vivo* (whole porcine liver) (Sano et al., 2010) to produce decellularised areas of tissue. *In vivo,* the host immune response clears cellular debris from the PEF treatment site to produce the decellularised scaffold (Phillips et al., 2010). But in the treatment of *ex vivo* or other biological samples, a clearing step must occur. Because of their solution-based nature, chemical decellularisation methods also facilitate ‘washing out’ of cellular debris as it is produced. As a decellularisation technique, PEF would need to be paired with a secondary washing step, whether it be chemical treatment, agitation or otherwise, to remove the remaining cellular material. Pore formation in cells of the treated tissue may allow for chemical treatment time to decrease, but there have been no studies assessing this effect to our knowledge.

In other applications, PEF was applied to modify physical or mechanical features of the biomaterial (Table 3). This technology was applied to liquid bioinks during printing to alter mechanical parameters of sodium alginate droplet, such as size and viscosity, as well as the tensile strength of composite zein, chitosan and poly (vinyl alcohol) films (Giteru et al., 2020; Rahman et al., 2021).

Sterility of the resulting scaffold is also critical to make it suitable for cell seeding and implantation. Studies have shown that PEF treatment can be utilised as a method of biomaterial sterilisation where heat, chemicals or UV-irradiation may not be desired to avoid degradation, unwanted structural changes, or cross-linking (Table 3) (Griffiths et al., 2008; Smith et al., 2009; Lerouge and Simmons, 2012).

## 6 The effect of PEF parameters on outcomes

### 6.1 Electrical field strength

The electrical field strength, *E,* is one of the key factors determining the outcome following PEF treatment and is a culmination of the many independent parameters involved in PEF. This factor can be calculated as follows:

$$Ε\;\left(\text{kV}/\text{cm}\right)=\frac{voltage\;delivered\;\left(U\right)}{width\;between\;electrodes}$$

This key factor, *E,* must be considered in relation to the desired outcome. As it is dependent on the width between electrodes, *E* will differ based on the PEF system and chamber used to deliver stimulation. As previously discussed, the transition between reversible electroporation and irreversible electroporation is threshold-dependent, based on the critical *E* reached (Edd et al., 2006; Lee et al., 2010). Above this critical threshold, cell viability is compromised, with permanent pore formation triggering apoptotic processes within the treated cells (Ganeson et al., 2018). Stronger electroporation at higher *E* can lead to enhanced extraction of intracellular compounds from the target cell.

The *E* applied to cell monolayers has been varied (Table 2). Electric field strengths of 0.3 kV/cm (pulse duration 5 m, frequency 1 Hz) and 0.8 kV/cm (pulse duration 100 µs, frequency 1 Hz) were cytotoxic to human dermal fibroblast populations *in vitro* (Gouarderes et al., 2020). A reduced *E* of 0.05 kV/cm (pulse duration 5 m) improved fibroblast migration in a wound scratch assay, but not to a statistically significant level (Gouarderes et al., 2020). Gouarderes *et al* did note that PEF-treated cells increased their expression of connective tissue growth factor (CTGF), VEGF-A, platelet-derived growth factor (PDGF)-A, and transforming growth factor (TGF)-α and reduced expression of TGF-β, and conditioned media from treated cells was able to stimulate the migration of unexposed cell populations (Gouarderes et al., 2020). The authors suggested that the enhanced expression of stimulatory factors by treated cells was due to release into the media during cellular necrosis, and cited mitochondrial stress as the cause of cell death rather than irreversible electroporation (Gouarderes et al., 2020). Das *et al* noted proliferative and migratory fibroblast responses after treatment of cells with 500 V (*E* not stated) (Das et al., 2020). In contrast to Gouarderes *et al.,* Liu *et al* noted that an *E* of 0.003 kV/cm was not cytotoxic to mouse fibroblasts embedded in a collagen lattice (Liu et al., 2018).

Griffiths *et al* treated *E. coli*-contaminated collagen gels with PEF stimulation and successfully inactivated *Escherichia coli* at densities below 10^3^ CFU/mL (Griffiths et al., 2008) (Table 3). They noted that the effect was increased with increasing *E* (from 12 to 45 kV/cm). A subsequent study noted that treatment of the collagen gels with up to 60 kV/cm had no effect on collagen gel structure or the growth of osteoblasts on these scaffolds (Smith et al., 2009). PEF treatment of small microbial cells generally requires high *E* (10-14 kV/cm) to reach the threshold for irreversible electroporation (Bhat et al., 2019). In comparison, Giteru *et al* assessed the effect of PEF stimulation on the characteristics of a film composed of zein, chitosan, and poly (vinyl alcohol) (ZCP), focusing on phenotypic outputs (Table 3) (Giteru et al., 2020). Treatment of film-forming dispersions with electric field strengths of 0.9–3.4 kV/cm (pulse width 32 µs, frequency 10–220 Hz) produced different particle sizes, viscosities and loss moduli depending on *E*. Particle size increased with increasing *E*, while viscosity and loss modulus decreased. An optimal tensile strength was achieved using an *E* of 3.4 kV/cm and 50 Hz frequency (Giteru et al., 2020). In this case, Giteru et al (2020) achieved their desired biomaterial phenotype with a much lower *E* of 3.4 kV/cm and did not require the high *E* needed for sterilisation. When considering the development of skin and other tissue constructs, biomaterials must be both sterile and have the appropriate mechanical features. It therefore needs to be considered that *E* may need to change based on the stage of biomaterial preparation and that if these stages are being executed simultaneously, this may be difficult based on changing PEF requirements.

Modifying *E* may provide control over cell behaviour or viability, but this parameter differed largely in these experiments. More systematic testing is required to elucidate the exact effect that *E* may have on cells both in 2D cell culture and in 3D engineered tissues.

### 6.2 Pulse shape

The shape of the pulses delivered by PEF can be exponentially decaying, sinusoid, or square, and can be monopolar or bipolar (Novickij et al., 2022). The choice of waveform can dictate the outcome following PEF, including whether electroporation is reversible or irreversible. Sinusoid and exponential pulses occupy time in the transient phases of the wave (i.e., increasing and decreasing), whereas square pulses rapidly reach and maintain their peak before rapidly decreasing. Given that PEF is threshold-dependent, square pulses are more effective at maintaining the electric field above the critical electrical field strength threshold compared to sinusoidal or exponential pulses. Therefore, square waves are much more effective at inducing irreversible electroporation compared to sinusoidal or exponential wave patterns (Novickij et al., 2022).

In addition to shape, the width of the pulse (τ_p_) is also a variable that can affect PEF outcomes. Obtaining a square pulse shape is easier with longer pulse durations, and pulse durations below 50 ns may compromise the square shape of the pulse, which can defect to a sinusoidal shape (Novickij et al., 2022). To maintain a square pulse shape, thereby maximising the effectiveness of PEF treatment, pulse duration should be considered.

Compared to unipolar pulses, bipolar pulses are more efficient at inducing electroporation (Kotnik et al., 2001b). Bipolar square pulses also mitigate the electrolytic contamination due to release of metal ions from the electrodes during PEF (Kotnik et al., 2001a). Substantial electrolytic contamination was observed when using unipolar square pulses and this has been linked to decreased cell viability and function (Loomis-Husselbee et al., 1991; Stapulionis, 1999; Kotnik et al., 2001a). Therefore, when developing tissue constructs, care should be taken to select appropriate pulse shape and polarity to avoid unwanted cytotoxicity.

In the preparation of biomaterials with PEF, much of the literature neglected to comment on their choice of pulse shape, however those that did, most commonly reported the use of rectangular or square pulses (Griffiths et al., 2008; Smith et al., 2009; Sano et al., 2010; Giteru et al., 2020) (Table 3). These studies failed to comment on the polarity of the selected pulses. Use of PEF for cell treatment was much the same, with the common choice being square or rectangular pulses (Table 2) (Hess et al., 2012; Kumar et al., 2016; Nguyen et al., 2018; Gouarderes et al., 2020; Gouarderes et al., 2022), with the occasional comment on polarity (Fitzsimmons et al., 2008).

### 6.3 Pulse number, duration, and frequency

The number of pulses delivered (*n*_p_), the pulse duration, and the frequency of pulse delivery can also affect PEF outcomes. Pulse number and duration impact the overall treatment time, thereby influencing the total energy input. Shorter pulse durations are generally preferred where cell viability needs to be conserved, while longer pulse durations aid the transfer of larger macromolecules across cell membranes (Rols and Teissié, 1998; Šatkauskas et al., 2012). The pulse frequency will affect the degree of pore formation and membrane permeabilisation (Lamberti et al., 2015; Novickij et al., 2022). Increased pulse frequency also augments the uptake of molecules by the targeted cells due to electrosensitisation, which should be considered in regards to the purpose of PEF treatment, for example, with drug delivery in electrochemotherapy (Bilska et al., 2000). This may be of assistance when combining PEF with delivery of pro-healing growth factors or drugs to cells or tissues (Berry-Kilgour et al., 2021). It has also been reported that pulse frequency influences the size of the pores formed during irreversible electroporation (Mi et al., 2019), which may influence the extraction of biological molecules from cells following PEF treatment.

Rahman *et al* noted that PEF-assisted electrohydrodynamic bioprinted sodium alginate droplets were altered by changing PEF parameters (Table 3) (Rahman et al., 2021). Using a modified continuous-style PEF delivery to bioink at pulse frequencies between 150 and 170 Hz, they reported that increasing frequency reduced the size and distance between droplets, but also reduced the consistency of droplet shape. Altering these parameters changed the printing outcome and should be considered based on the desired product.

Pulse number and frequency were also shown to influence decellularisation of structures (Table 3) (Phillips et al., 2010; Sano et al., 2010). Phillips *et al* applied 90 pulses at 1 or 4 Hz (*E* = 1.75 kV/cm) to rat carotid arteries *in vivo* by applying an electrode clamp to either side of the artery (Phillips et al., 2010). Five days after PEF treatment, they noted a loss of cells and decrease in nuclear staining in histological sections. Apoptotic cells resulting from irreversible electroporation were cleared by host immune responses to produce an acellular vessel. In the 1 Hz-treated vessels this effect was sustained beyond 7 days, but in the 4 Hz-treated vessels, repopulation of cells into the vessel was noted by day 7 (Phillips et al., 2010). Sano *et al* produced visible depigmented lesions on *ex vivo* porcine livers following PEF treatment with similar parameters (99 pulses, 0.25–4 Hz, *E* = 1 kV/cm, pulse width = 100 µs). Histological analysis of these lesions showed reduced cell number and preserved extracellular matrix structure, but no significant markers of decellularisation such as DNA content or DAPI staining were evaluated (Sano et al., 2010).

The effect of changing these PEF parameters on cells has not been extensively investigated. For the skin cell studies (Table 2), frequencies used were around 1 Hz. The effect of pulse number, duration and frequency on standardised cell outputs such as cytotoxicity or proliferation has rarely been reported in the literature. While standardised use of these outputs would aid comparison, functional assessments of cell behaviour have differed based on the cell type, for example, alignment of fibroblasts, or migration of keratinocytes. Liu *et al* found that increasing pulse frequency from 0.0002 up to 100 Hz increased fibroblast alignment in a collagen matrix (Liu et al., 2018). In another study, changing frequency from 0.1 to 1,000 Hz (*E* = 150 mV/mm, other parameters not stated) did not affect the migration capacity or speed of keratinocytes *in vitro* (Ren et al., 2019). Pulse duration also impacted fibrotic responses in cultured dermal skin substitutes (Gouarderes et al., 2022), where a long pulse protocol (10 pulses of 5 ms) increased MMP activity and reduced TGF-β1 expression to a greater extent than a short pulse protocol (8 pulses of 100 µs). Although, a transient decrease in collagen content was observed regardless of the protocol used (Gouarderes et al., 2022).

### 6.4 Sample conductivity and temperature

A highly conductive sample is at greater risk of arcing, a phenomenon where high *E* results in electrons jumping from one electrode to the other, rather than moving through the sample (Góngora-Nieto et al., 2003; Álvarez et al., 2006). This arcing decreases the efficacy of electroporation, increases sample temperature, and can cause damage to the electrodes (Barbosa-Cánovas et al., 1999; Álvarez et al., 2006; Oey et al., 2022). Because conductivity will determine the efficiency of PEF, the conductivity of chosen biomaterials needs to be considered. More conductive biomaterials (>4 m/cm) are showing promise in the engineering of electrically-responsive cardiac and muscle tissues (Gajendiran et al., 2017; Dong et al., 2020). Because high conductivity is linked to reduced efficiency, it may be easier to prevent arcing and loss of electroporation efficiency when incorporating PEF into the engineering of tissues such as epithelium or skin, which have lower conductivities (Saberi et al., 2019).

Temperature will increase following irreversible PEF treatment because of Joule heating (Davalos and Rubinsky, 2008; van Gemert et al., 2015). At higher temperatures, sample conductivity increases, which increases the likelihood of arcing (Barron and Ashton, 2005). In addition to considering success of PEF treatment, the desired outcome following PEF treatment should also be considered when optimising temperature. Increased temperature can enhance electroporation efficiency, but at the detriment of the target tissue, with a higher level of tissue damage and disintegration (Lebovka et al., 2005).

Whether or not the structure of the tissue construct must be maintained during and after PEF treatment should be evaluated when considering the sample temperature. For example, for the purpose of sterilisation or decellularisation of biomaterials, maintaining the appropriate structure is critical for success. Pre-sterilisation of temperature-sensitive collagen-based biomaterials were performed using cooling systems to reduce temperature and mitigate structural changes (Griffiths et al., 2008; Smith et al., 2009). Classical PEF studies have typically been conducted under chilled conditions or at room temperature (≤25°C) (Leong et al., 2014; Liu et al., 2017; Kantono et al., 2021; Karki et al., 2023). Complications may arise with PEF treatment of cell suspensions or 3D constructs, which must be maintained at physiological temperature. Liu *et al* looked at the effect of PEF on fibroblasts embedded within a collagen matrix at physiological temperature (37°C), but did not comment on the effect on the collagen matrix (Liu et al., 2018) (Table 2). It is well-known that collagen is temperature-sensitive (Miles and Bailey, 1999), and the increased sample temperature following PEF should be considered as it could affect the degradation rate not only of collagen, but of other physiologically relevant biomaterials. This becomes particularly important when looking ahead to PEF treatment of 3D tissue constructs and their stability *in vivo* or for prolonged culture periods at physiological temperature.

## 7 Conclusion and future directions

**FIGURE 3:**
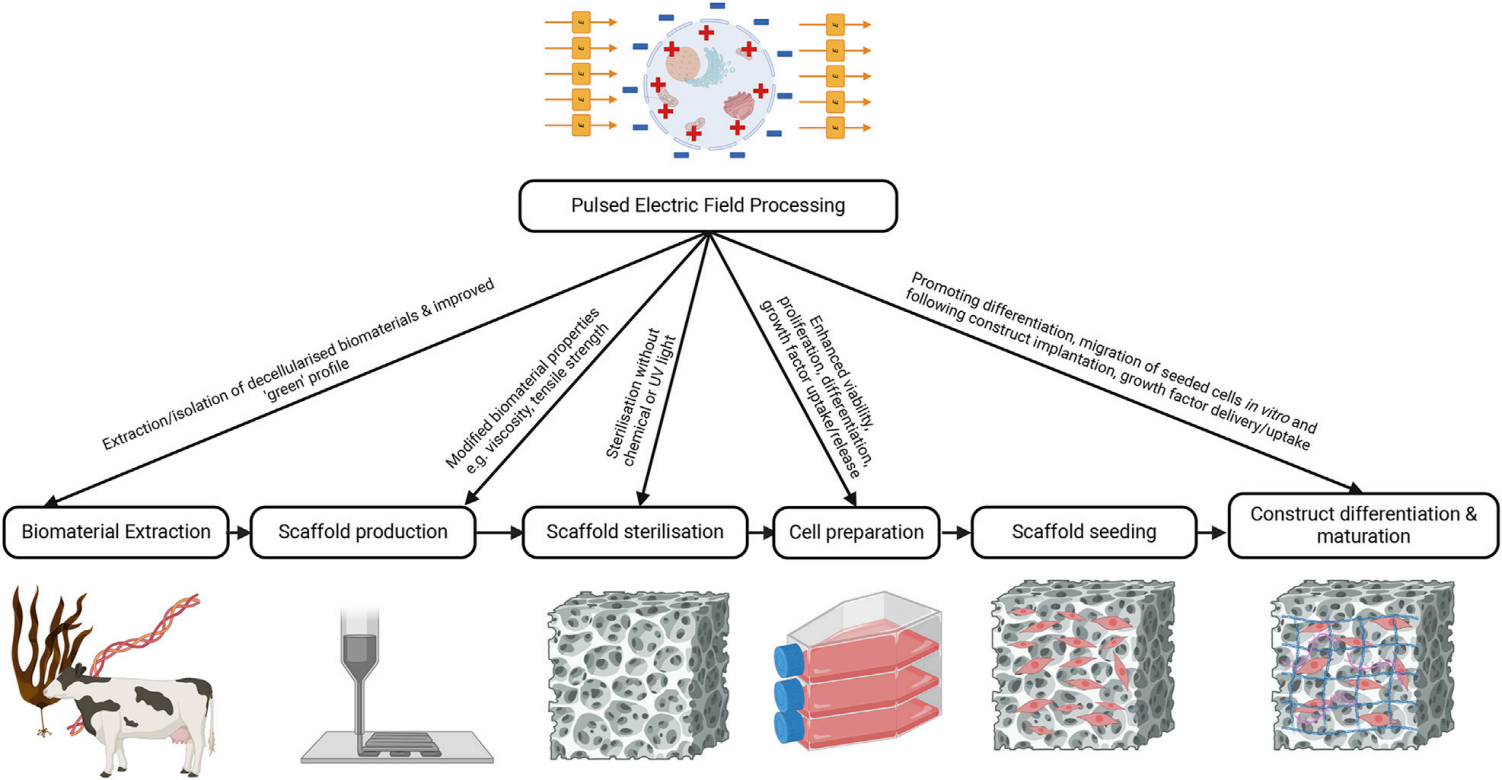
Integration of PEF into skin engineering. Pulsed electric field processing may be able to enhance the extraction of biomaterials and do so in a more sustainable manner compared to current practices. It may also be able to be used to alter biomaterial properties during 3D printing or assist with isolation of decellularised matrices. PEF may also be useful in the sterilisation of biomaterial constructs to make them compatible for cell seeding. Finally, PEF could be used to modify cell behaviour by promoting differentiation, migration, or proliferation of seeded cells to encourage success of the tissue construct. Created with BioRender.com.

It is evident from the literature discussed that PEF has great potential to increase the reparative function of skin cells, aid in the preparation of biomaterials, and accelerate the repair of skin wounds *in vivo*, if appropriate PEF processing parameters are applied. Inconsistent reporting of parameters was noted across studies, which limited comparisons, and may reduce the capacity of others to replicate this work. It is still unclear how PEF will be best applied to the engineering of skin and other tissues. The evidence to date suggests that PEF may fit into multiple points along the process to provide a scalable, sustainable additive to current engineering practices (Figure 3). These steps may include the preparation of biomaterials including extraction, isolation of decellularised tissues, control of scaffold structure during reconstitution, as well as scaffold sterilisation prior to cell seeding. In addition, PEF may be used to promote proliferation or differentiation of cells during their expansion prior to seeding into a scaffold, or in developing mature tissue constructs post-seeding (Figure 3). It may directly facilitate cell and tissue growth or indirectly enhance the production or delivery of growth factors and signalling molecules.

The application of PEF to skin cells has had variable and often cell-type specific effects, thus its effects need to be elucidated in such a way that accounts for changes in the cell populations and functions throughout the dermis, epidermis, and hypodermis. It will be important to distinguish whether the effects of PEF on particular cell types are due to electrotaxis or electroporation. While there is evidence linking application of electric fields, particularly changes in *E*, to cellular migration and proliferation, understanding as to how electroporation of skin cells affects their differentiation state and expression of signalling molecules is sparse. It also must be elucidated how the use of different PEF generators and chambers may impact delivery of current through cellular samples, and the downstream effects these might have.

At this stage, PEF is an underutilised resource in skin engineering. Future studies should be designed to drive decision-making moving forward about how best to apply PEF to different skin cells, biomaterials, and/or engineered skin constructs to enhance their therapeutic application to skin burns and wounds. Systematic investigations linking PEF input parameters to a traceable or modifiable biological outcomes will be key to the integration of PEF into current tissue engineering approaches.
